# Dealing with Dust Entrained in the Nitrogen Plume Demonstration

**DOI:** 10.1021/acs.jchemed.3c00711

**Published:** 2023-09-29

**Authors:** Dean J. Campbell, Q. Ott, Thomas S. Kuntzleman

**Affiliations:** † Mund-Lagowski Department of Chemistry and Biochemistry,Bradley University, Peoria, Illinois 61625, United States; § Department of Chemistry, Wayne State University, Detroit, Michigan 48202, United States

**Keywords:** General Public, First-Year Undergraduate/General, Demonstrations, Analogies/Transfer, Liquid Nitrogen, Iron, Safety

## Abstract

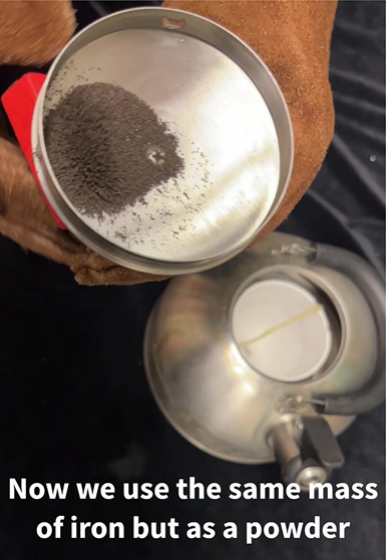

Water condensation plumes produced by the addition of iron powder to liquid nitrogen can be contaminated with small quantities of particulate matter. Variations on the plume demonstration, including those using noisemakers, are described to help minimize the release of particulates into the air.

## Decreasing Dust

Water condensation plumes can be produced by adding iron spheres to liquid nitrogen in a vented container such as a plastic milk jug, as described in a recent paper in this *Journal*.^1^ When the spheres at room temperature transfer their energy as heat to liquid nitrogen, the cold nitrogen vapor escapes the vent and condenses water vapor in the surrounding air to make the small liquid water droplets that make the plume visible. In addition to the visual appeal of a plume of fog, these demonstrations illustrate the relationship between surface area and kinetics of processes.^2−5^ Demonstrations such as these have been shown to a variety of audiences, including school children, often as part of a group of liquid nitrogen-based activities. In addition to the surface area/kinetics connection, the demonstrations reported here can be used to discuss heat transfer and phase changes.

To produce larger plumes, liquid nitrogen in the container must boil more rapidly as a result of faster heat transfer from the solid iron into the liquid. One way to achieve faster heat transfer is to increase the surface area of iron in contact with the nitrogen. This can be shown by comparing the plumes produced using two samples of iron with the same mass, in which one sample is divided into a few spheres and the other is divided into many smaller particles. The more finely divided samples have larger surface area and will therefore boil the liquid nitrogen more quickly to produce larger condensation plumes.^1^ For example, in a previous paper in this *Journal*, 33 g samples of metallic iron were added to liquid nitrogen in the form of 4 half-inch (12.7 mm) diameter spheres, ∼32 quarter-inch (6.4 mm) diameter spheres, ∼256 eighth-inch (3.2 mm) diameter spheres, or iron powder. Samples composed of smaller spheres or particles of iron had greater surface area and produced larger plumes more quickly. Metallic iron will oxidize in contact with the oxygen in the air, but even spheres with rusty surfaces will show the influence of iron surface area on plume size. (Note that very small, pyrophoric iron particles were not part of these studies.)

This demonstration of surface area is a nice way to produce plumes containing small quantities of solid particulates. This is in contrast to the previously reported “magic genie” plumes of water condensation that were produced by adding manganese(IV) oxide powder to a solution of hydrogen peroxide. In this case, aerosolized manganese(IV) oxide and hydrogen peroxide could be entrained in the plumes.^6^ Unfortunately, we have observed that small solid particles of iron or iron oxides can be released with nitrogen-based plumes in certain cases. If sufficient numbers of particles are entrained in the plume, they can turn the emission to a gray or orange color. This is a possible inhalation hazard to both the people running the experiment and those observing it. There are multiple ways to address these problems with the liquid nitrogen-based plumes:

- Do not use iron granules or powder; only use large and small iron spheres. The advantage is that the spheres can have a well-defined surface area, but they could still carry loose rust particles into the liquid nitrogen.
- Clean the iron with water or acetone in order to rinse away small rust or iron particles. The iron should be quickly dried after it has been wet for any reason in order to help prevent new rust from forming.
- Deflect the plume downward to redirect small particles downward. Of course, this approach seems to defeat the purpose of making as large of plumes as possible, but an alternative approach is described below.

## Making Noise Instead of Plumes

**Figure 1:**
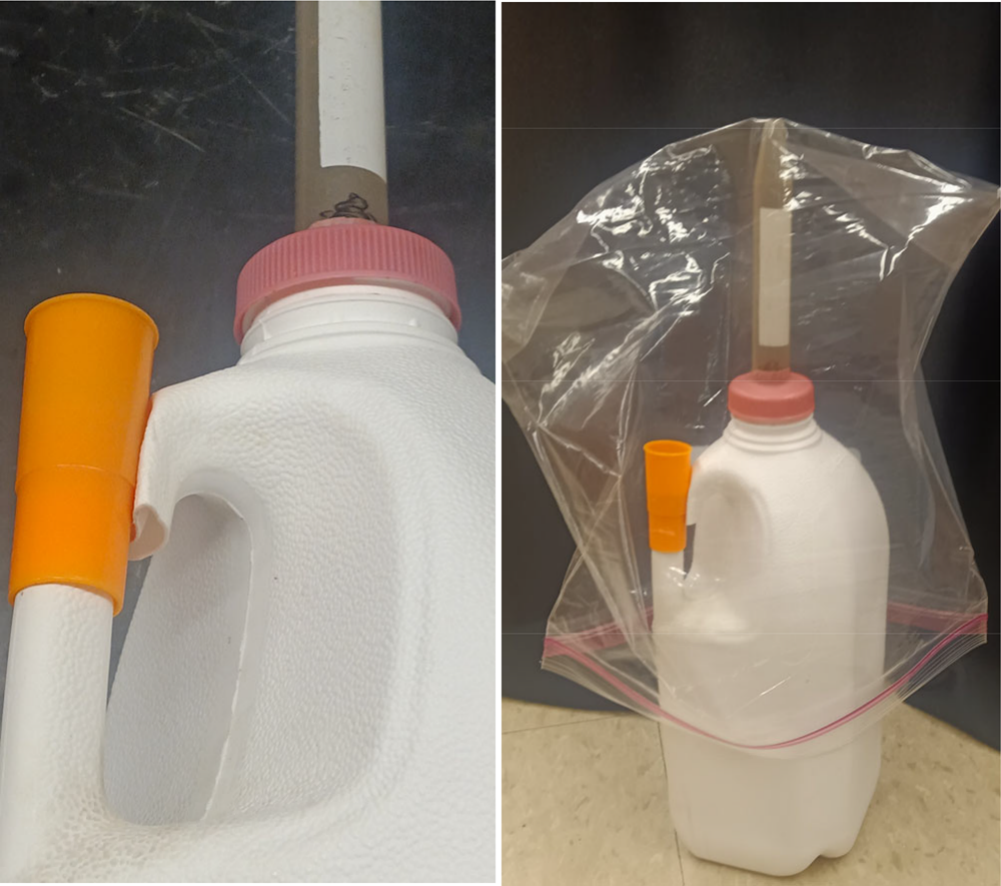
Plastic container (milk jug) with handle cut through and siren whistle added. The centrifuge tube dropping mechanism is seen protruding through the pink bottle cap.

An alternative to the visual observation of condensation plumes from the vent of the container containing liquid nitrogen is to place an anemometer over the bottle vent.^1^ A less sophisticated but more entertaining approach involving a noisemaker is shown in Figure 1. To construct the assembly, the handle of a half gallon plastic milk jug was cut completely through perpendicularly to the orientation of the handle. A plastic siren whistle was placed over the lower sliced end of the handle. The siren whistles had an internal diameter of approximately 14 mm and were purchased from the US Toy Company, Grandview, MO.^7^ The upper sliced end of the handle was pushed inward along one side to accommodate the siren whistle, Figure 1 left, but still provided an extra vent for excessive nitrogen gas pressure. In this demonstration, we poured liquid nitrogen into the bottom of the milk jug until it was about 1/8 full. The same dropping mechanism from the previously described demonstration was used: a lid from a milk jug with a centrifuge tube pierced through it, as shown in Figure 1.^1^ The tube was filled with about 33 g of iron spheres or powder, and a magnet held the iron in place by placing it outside the tube so the jug lid could be screwed on; then, the magnet was removed to drop the iron into the nitrogen to sound the whistle.

When the nitrogen gas flowed through the whistle sufficiently fast, the noisemaker produced a rather silly revving sound. Flow rates that were too slow did not produce noise. A plastic bag could be placed over the whistle to deflect the plume and potential solid particles downward. A YouTube video showing the whistle assembly in action is available.^8^ Large spheres added to liquid nitrogen caused it to boil, but the rate of nitrogen gas production was too low to sound the whistle. A similar mass of iron in the form of iron filings caused the liquid nitrogen to boil sufficiently hard to sound the whistle. The plastic bag placed over the whistle fogged up with condensation but did not prevent the sounding of the whistle.

The container with a spout bearing a noisemaker was reminiscent of a tea kettle shape, which inspired attempts to extend these experiments to an actual tea kettle. To do so, a polystyrene foam cup was cut down in height to enable it to be placed into a tea kettle with a lid in place. The foam cup was removed, filled with liquid nitrogen, and returned to the tea kettle. The cup did not shrink sufficiently in contact with the liquid nitrogen to present a problem. The foam cup insulated the rest of the kettle from the cold, slowing the rate of vaporization of liquid nitrogen and helping to prevent the kettle from frosting up. A single 8.3 g iron sphere was held inside the kettle lid with a magnet; the lid, magnet, and sphere were placed on the kettle, and the magnet was removed to release the sphere into the liquid nitrogen. The rate of boiling was too low to cause the tea kettle to whistle. On the other hand, repeating this experiment using the equivalent mass of iron as a powder rather than a single sphere produced a greater rate of boiling, which caused the kettle to whistle. Similar to the bag described above, a lab wipe placed above the vent hole could be used to collect dust entrained in the nitrogen plume. A YouTube video showing the tea kettle assembly in action can be found online.^9^

Horns and whistles vary considerably in their design, so some trial and error might be required to find the right noisemaker for these demonstrations. Given that the visibility of a condensation plume can depend on the humidity of the surrounding air, the noisemaker approach is a compelling way to show this demonstration to audiences, regardless of environmental conditions.

## Safety

Plastic can become brittle at low temperature, and a safety shield is recommended whenever using liquid nitrogen. Make sure all containers that contain liquid nitrogen are properly vented; NEVER seal up a container with liquid nitrogen inside. The noisemaker must be able to easily make noise with sufficient gas flow but also must not prevent nitrogen gas from leaving the container. The noisemaker must allow the container to adequately vent and not over pressurize with nitrogen gas. Use a shield when working with liquid nitrogen. Proper personal protective equipment such as goggles must be used, ESPECIALLY considering that vertical plumes of materials are produced from potentially cold-embrittled materials. Avoid spilling reagents on clothing. Avoid skin contact and wear insulating gloves while working with liquid nitrogen or working with objects that have been cooled by liquid nitrogen. Always wash hands after completing the demonstrations.
